# Nrf2 Modulation in Breast Cancer

**DOI:** 10.3390/biomedicines10102668

**Published:** 2022-10-21

**Authors:** Somayyeh Ghareghomi, Mehran Habibi-Rezaei, Marzia Arese, Luciano Saso, Ali Akbar Moosavi-Movahedi

**Affiliations:** 1 Institute of Biochemistry and Biophysics, University of Tehran, Tehran 1417466191, Iran; 2 School of Biology, College of Science, University of Tehran, Tehran 1417466191, Iran; 3 Center of Excellence in NanoBiomedicine, University of Tehran, Tehran 1417466191, Iran; 4 Department of Biochemical Sciences “A. Rossi Fanelli”, Sapienza University of Rome, 00185 Rome, Italy; 5 Department of Physiology and Pharmacology “Vittorio Erspamer”, Sapienza University of Rome, 00185 Rome, Italy; 6 UNESCO Chair on Interdisciplinary Research in Diabetes, University of Tehran, Tehran 1417466191, Iran

**Keywords:** oxidative stress, antioxidant pathway, Keap1-Nrf2, breast cancer, Nrf2 inhibitors

## Abstract

Reactive oxygen species (ROS) are identified to control the expression and activity of various essential signaling intermediates involved in cellular proliferation, apoptosis, and differentiation. Indeed, ROS represents a double-edged sword in supporting cell survival and death. Many common pathological processes, including various cancer types and neurodegenerative diseases, are inflammation and oxidative stress triggers, or even initiate them. Keap1-Nrf2 is a master antioxidant pathway in cytoprotective mechanisms through Nrf2 target gene expression. Activation of the Nfr2 pathway benefits cells in the early stages and reduces the level of ROS. In contrast, hyperactivation of Keap1-Nrf2 creates a context that supports the survival of both healthy and cancerous cells, defending them against oxidative stress, chemotherapeutic drugs, and radiotherapy. Considering the dual role of Nrf2 in suppressing or expanding cancer cells, determining its inhibitory/stimulatory position and targeting can represent an impressive role in cancer treatment. This review focused on Nrf2 modulators and their roles in sensitizing breast cancer cells to chemo/radiotherapy agents.

## 1. Introduction

The oxidative stress (OS) condition is defined as an imbalance between the generation and elimination or scavenging of the reactive oxygen species (ROS) or reactive nitrogen species (RNS). The latter involves various cellular mechanisms, including defensive antioxidant systems [1]. A high level of ROS creates an unfavorable condition for normal cells and leads to cell damage by harming DNA, proteins, and various lipids in the cytoplasm or cell membrane [2]. Furthermore, the high level of ROS promotes tumorigenesis through various biomolecular modifications. Therefore, cells utilize various antioxidant systems to moderate these conditions and avoid their destructive effects. In cancer, ROS also plays a dual role as the activator of the transformation of a normal cell to oncogenic cells while rolling in tumor inhibition [3]. Keap1-Nrf2 is a master antioxidant pathway in cells that reduces ROS and provides suitable conditions for cell growth and proliferation. Activation of Keap1-Nrf2 is a leading regulatory pathway in counteracting ROS and its harmful effects [4,5]. Nuclear factor-erythroid-2-related factor 2 (Nrf2) plays a pivotal role in the basal activity and the corresponding induction of genes encoding several antioxidant and phase II detoxifying enzymes and related proteins. Nrf2 is present in the cytoplasm as an inactive complex through binding with a repressor molecule known as Keap1 (Kelch-like ECH-associated protein 1), which assists its ubiquitination. Keap1 contains several reactive cysteine residues as sensors of intracellular redox conditions. Oxidative or covalent modification of thiols in some of these cysteine residues results in the activation of the Nrf2 pathway and triggers various genes’ expressions, which are involved in creating balanced oxidative conditions [6]. Besides the cytoprotective role of this pathway, Nrf2 can be expressed indefinitely in cancer cells through the gain of mutations [7]. Its hyperactivation accounts for the oncogenic feature of cancer cells and triggers cancer cell growth, proliferation, angiogenesis, and chemo/radioresistance.

**Figure 1:**
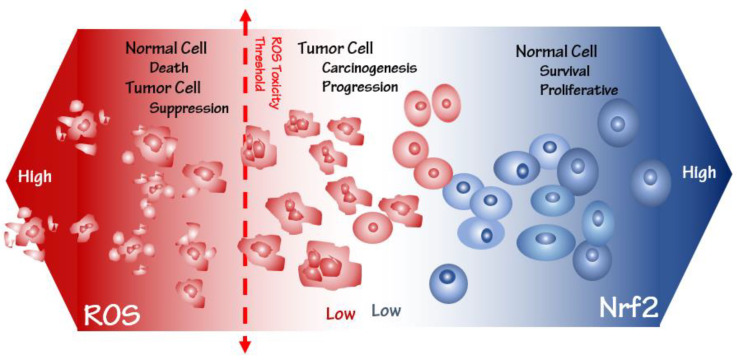
Nrf2 and ROS levels determine cell fate propensity. ROS at a higher toxicity level brings about normal cell death and tumor cell suppression, while at a lower toxicity level, it is associated with carcinogenesis and tumor progression. Conversely, high-level Nrf2 survives normal cells against ROS-related cancer progression.

Hyperactivation of Nrf2 involves cell survival and proliferation through alterations in cell metabolism and functional adaptations, despite cells’ normal and cancerous state. Therefore, Nrf2 targeting cancer progression can provide a new perspective in designing more effective drugs. Breast cancer is the most common cancer in women worldwide, accounting for approximately 2.3 million new breast cancer cases and 685,000 deaths in 2020. The cases are estimated to reach 4.4 million in 2070 [8,9]. Various risk factors are involved in breast cancer incidence, including sex, aging, estrogen, family history, gene mutations, and unhealthy lifestyle [10,11]. According to several studies, disrupting the essential anti-oxidant system at a high level of ROS (but lower than the toxicity threshold) in cells can trigger the incidence and progression of various cancer [12,13,14]. However, ROS at a level higher than the toxicity threshold involves cell death and cancer suppression. Therefore, precisely identifying molecular mechanisms involving Nrf2 expression and regulation is helpful in cancer treatment [15]. To deal with the effects of ROS, cells modulate several signaling pathways, which leads to the creation of optimal conditions for their unlimited growth and proliferation. Hyperactivation of the Nrf2 pathway in cancer cells provides a suitable condition for their growth, proliferation, and drug resistance due to decreasing ROS lower than the toxicity threshold. Thus, the downregulation of this pathway by various inhibitors to raise the level of ROS can suppress cancer cell development. In this review, we focused on various functions of the Nrf2 pathway, including a comprehensive explanation of its role in the expansion of cancer cells, and introduced some inhibitors of this pathway into the treatment of breast cancer and related studies (Figure 1).

## 2. Oxidative Stress-Related Inflammatory Pathways

The term oxidative stress was used in 1985 to describe specific cellular conditions [16]. In normal condition, cells can eliminate free radicals and reactive metabolites such as ROS and RNS, which is generated as a result of cellular metabolism. If the amount of these active species is more than the antioxidant capacity of the cells, this balance will be disturbed. This disturbance in the process of removing active species has led to the creation of an oxidative stress condition, which results in the destruction of biomolecules in the first stage. The second stage is the destruction of cells, which will finally destroy the entire living organism [16,17]. The biological reduction of molecular oxygen (O_2_) through endogenous mitochondrial respiratory chain reactions generates ROS, such as superoxide anion (O_2_^−^), hydrogen peroxide (H_2_O_2_), hydroxyl radical (OH^•^), and organic peroxides [18]. These products of a healthy cellular metabolism display a pivotal effect in triggering various signaling pathways in cells in dealing with changes in cell conditions [19,20]. Under dysregulated and disturbed oxidative stress conditions, the imbalanced antioxidant capacity of the cells damage biomolecules to increase carcinogenesis risk [21,22]. A range of transcription factors can be activated by oxidative stress in cells, such as nuclear factor kappa light chain enhancer of activated B cells (NF-κB), hypoxia-inducible factor 1α (HIF-1α), activator protein 1(AP-1), c-Myb, p53, peroxisome proliferator-activated receptor γ (PPAR-γ), β-catenin/Wnt, and nuclear factor erythroid 2 [NF-E2]-related factor 2 (Nrf2). The activation of various transcription factors can lead to the expression of many genes that are involved in different aspects of cellular mechanisms. Therefore, the production of ROS over the antioxidant capacity of cells can induce specific pro-inflammatory pathways [20,23,24].

## 3. Oxidative Stress Susceptibility of the Cancer Cells

The production of ROS and RNS is a common event in cellular metabolism and occurs in all cells. In conditions below the threshold, these active species act as stimulators of some signaling pathways and initiate downstream cascades [25]. In addition, cells have a complete set of direct and indirect anti-oxidant systems to inhibit the harmful effects of these species. The oxidative stress condition induces upregulation of the anti-oxidant system to maintain a balance between producing and removing ROS/RNS. The susceptibility of cellular constituents to oxidative stress depends on the redox status, which is controlled by the levels of ROS and local anti-oxidant defense capacity. In imbalanced conditions, oxidative stresses can trigger various damages to biomolecules and promote tumorigenesis. A high level of ROS is associated with numerous behaviors in the cancer cell, such as survival, proliferation, angiogenesis, and metastasis [25,26]. As mentioned in the previous section, the production of ROS over the anti-oxidant capacity of cells can induce specific pro-inflammatory pathways. In response to oxidative stress, phosphorylation of the inhibitor of NF-kB (IKB), as an inhibitory protein of the NF-kB pathway, leads to the activation of a variety of NF-kB-related genes involved in significant processes, such as inflammation, tumor invasion, and angiogenesis [27,28,29]. On the other hand, oxidative stress can control the expression and activity of HIF-1*α* through different signaling factors, such as the prolyl hydroxylase domain (PHD)-containing protein, PI3K, and some microRNA. Thus, oxidative stress is involved in inflammation, the epithelial–mesenchymal transition (EMT), and extracellular matrix deposition mediated by HIF-1 via cooperating with the NF-kB pathway [30,31]. AP-1 is a critical nuclear transcription factor in that its activity can be controlled through increased synthesis of its components and their phosphorylation. This transcription factor is connected to JNK, the p38 MAP kinase (MAPK) cascades through its components, and their activities are promptly increased in response to the exposure to various pro-inflammatory stimuli [32]. Overexpression of c-Myb as an oncogene molecule has been observed in several cancers. The c-Myb gene encodes an extremely conserved 78 kDa transcription factor involved in various biological manners, including regulating cell proliferation, differentiation, and apoptosis. The formation of excessive ROS can alter the c-Myb signaling pathway through its phosphorylation and activation [33]. The correlation between oxidative stress and p53 is one of the most important pathways in many biological processes. P53, as a guardian of the genome, plays a critical role in response to the oxidative condition. Interestingly, this molecule shows different responses in different conditions; in this way, it acts as an antioxidant molecule at a low level of oxidative stress and a pro-oxidant at a high level of oxidative stress, and with increasing stress, it leads to cell death [34]. Furthermore, the Wnt signaling pathway is one of the major targets of p53. The canonical Wnt/β-catenin pathway controls numerous signaling pathways in development and tissue homeostasis. Overexpression of various Wnt target genes such as c-Myc, cyclin D1, and HIF-1α is observed in the stimulation of this pathway, which is involved in the initiation and development of various cancers [35]. On the other hand, as mentioned, the overproduction of ROS leads to DNA damage, and p53 is one of the most important molecules in DNA repair. Furthermore, Nrf2 triggers the expression of p53 binding protein 1 (p53BP1) and protects cells against ionizing radiation-induced DNA damage and genome instability [36]. P53 can induce β-catenin degradation through its poly-ubiquitination [37]. The proliferator-activated receptor ɣ (PPARɣ), a member of the nuclear receptor superfamily, one of the most widely examined ligand-inducible transcription factors, shows essential performance in adipocyte differentiation, maintenance, and function, as well as the maturation of numerous immune cells including monocytes/macrophages, dendritic cells, and lymphocytes. Moreover, this transcription factor has a regulatory effect on cell propagation in several other tissues and organs, and dysregulation of this pathway is connected to the progression of tumors [38]. Based on some studies, PPARɣ can protect cells from ROS through the upregulation of Bcl-2 expression [39]. In addition to all the various pathways involved in the promotion/inhibition of inflammatory responses in oxidative conditions, Keap1-Nrf2 signaling is a master antioxidant pathway with very interesting properties. In oxidative conditions, the basic leucine zipper transcription factors, including Nrf1 and Nrf2, heterodimerize with sMaf family proteins and bind to the antioxidant response element (ARE) [40]. Activation of this pathway is one of the most significant mechanisms for achieving a high level of ROS and protecting cells from oxidative stress. Nrf2 downstream genes are involved in cell metabolism, intracellular redox hemostasis, apoptosis, and autophagy [41]. According to these explanations, Nrf2 has been offered as a tumor suppressor. On the other hand, over-activation or activation is observed in various diseases, including several types of multidrug-resistant (MDR) cancer, autoimmune, neurodegenerative, and cardiovascular diseases. Hyperactivation of Keap1-Nrf2 creates a context that helps the survival of healthy and malignant cells, defending them versus oxidative stress and chemo/radiotherapy agents. In this case, this pathway can be accepted as an oncogene, which can favor the conditions for the initiation and development of tumor cells [42,43]. In acute oxidative conditions, accumulating various damages is an effective stimulator of apoptosis [44]. They can trigger the intrinsic mitochondrial pathway, the extrinsic death receptor pathway, and the endoplasmic reticulum (ER) stress pathway [45,46]. Furthermore, ROS production is a key mechanism shared by chemo/radiotherapeutic approaches for cancers due to its association with triggering cell death [47,48]. Therefore, ROS has a dual function as a tumor suppressor and a tumor inducer. Based on certain findings, producing more ROS leads to irreversible oxidative stress, and these high levels of ROS stimulate cancer cell death. A high level of ROS is very unpleasant for cancer cells because it suppresses the chemotherapeutic resistance of cancer cells and increases their sensitivity to drugs. In addition, it triggers certain signaling pathways that lead to various cell death, including apoptosis, necrosis, and cancer cell autophagy [49,50,51]. To deal with irreversible oxidative stress, cells constantly upregulate various anti-oxidant pathways to keep ROS levels below the threshold of cell death stimulation and trigger chemotherapeutic resistance of cancer cells. As mentioned, the Nrf2 pathway is one of the main candidates for inducing the desired conditions for cancer cell growth and proliferation.

## 4. Keap1-Nrf2 as a Master Oxidative Stress Sensor and Its Cytoprotective Role

One of the most basic pathways involved in dealing with oxidative stress conditions is the Kelch-like ECH-associated protein 1 (Keap1)–nuclear factor erythroid 2-related factor 2 (Nrf2) pathway, which controls the transcription of various antioxidant genes that maintain cellular redox homeostasis through the elimination of carcinogens and various toxins [52,53,54]. The 624-amino acid Keap1 protein has three functional domains, including a general complex/tramtrack/bric-a-brac (BTB) domain, an intervening region (IVR), and a Kelch domain, also identified as the double glycine repeat (DGR) domain, as well as more than 20 free sulfhydryls (SH) groups in its constituent cysteine residues in IVR and BTB domains. These highly reactive functional groups act as stress sensors, and various oxidative stresses can modify these residues [55,56,57,58]. Two residues, Cys273 and Cys288, appear to be important for Keap1 to regulate Nrf2 under normal and stress conditions, while Cys151 is mainly essential in oxidative conditions [59,60,61,62]. Other Keap1 residues, including Cys226, Cys434, and Cys613, appear essential for detecting particular toxins. The BTB domain binds Cul3 and is necessary for Keap1 dimerization. Keap1 can attach to the N-terminal Neh2 domain of Nrf2 through its Kelch/DGR domain. BTB and Kelch domains are connected via the IVR domain and regulate the activity of Keap1 [7]. Nrf2, as a transcription factor, has 605 amino acids that are categorized into seven functional domains (Neh1-7). The Neh2 domain, situated in the N-terminal of Nrf2, is the main regulatory domain [63]. This domain comprises seven lysine residues accountable for ubiquitin conjugation and two binding sites (ETGE and DLG motifs) involved in Nrf2 stability [64]. The ETGE and DLG motifs interact with Keap1 as an adapter for the Cul3-Rbx E3 ubiquitin ligase complex, which suppresses Nrf2 by stimulating its ubiquitination and consequent proteasomal degradation [65,66,67]. The Neh1 and Neh6 domains have also been involved in regulating Nrf2 stability. CNC type bZIP DNA-binding motif in Neh1 is involved in Nrf2 connecting DNA and its dimerization with other transcription factors [68]. The Neh3, Neh4, and Neh5 domains interact with coactivators to enhance the transactivation of Nrf2 target genes. In the following, Neh7 can link with the retinoic X receptor α (RXRα), an Nrf2 inhibitor, and suppress Nrf2 target gene transcription [69]. Under normal conditions, Keap1 regularly targets Nrf2 for ubiquitin-dependent degradation to preserve low Nrf2 levels [70,71]. Based on the ‘hinge and latch’ model of the Nrf2/Keap1 interaction, each Kelch domain of a Keap1 homodimer connects to one Nrf2 protein through a weak-binding DLG motif (latch) and a strong-binding ETGE motif (hinge) situated in the N- terminal Neh2 domain of Nrf2 [64,72]. According to previous results, the binding affinity of Kelch for the ETGE motif is approximately 100-fold higher than for the DLG motif [73,74]. As mentioned, Keap1 can be linked to Nrf2 in normal conditions and induce its degradation by the proteasome. In contrast, in oxidative conditions, the separation of Keap1 from Nrf2 will lead to the activation of the Nrf2 pathway and upregulation of its target genes. Furthermore, some proteins, including p21 and p26, through the interference of the Keap1 and Nrf2 interaction, can increase Nrf2 gene expression. Oxidative stress can induce the upregulation of p26 gene expression and trigger Nrf2 target gene [75,76]. In addition to the leading role of Keap1 in controlling the Nrf2 pathway, a large number of various kinases such as protein kinase C(PKC), MAPK/ERK/JNK, JUN/MYC, and PI3K are involved in its regulation [77].

### Cytoprotective Role of Keap1-Nrf2 Pathway

**Figure 2:**
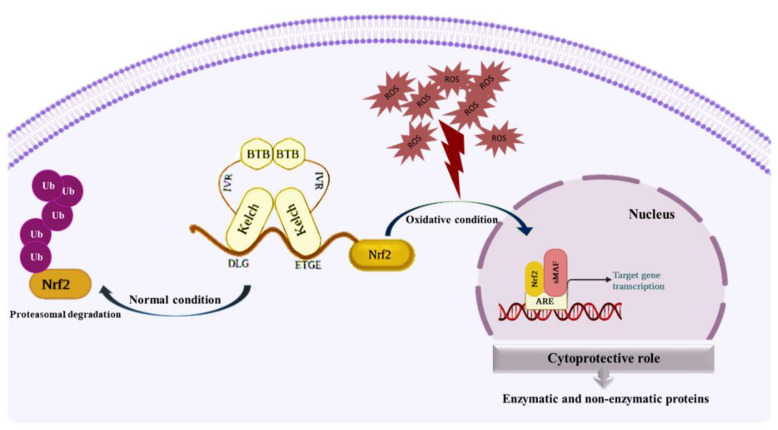
Cytoprotective role of Keap1-Nrf2. Under high ROS levels and oxidative conditions, Nrf2 translocates to the nucleus and triggers the expression of anti-oxidant proteins.

Excessive oxidative stress as a result of altering biomolecules disrupts cells’ normal function [77]. ROS can trigger various aspects of tumor cell initiation and progression: (a) Increased cellular proliferation through extracellular-regulated kinase 1/2 (ERK1/2) and ligand-independent RTK activation, (b) bypassing of apoptosis through activation of some specific signaling pathways such as PI3K/AKT and NF-kB, (c) increased metastasis and tissue invasion through overexpression of matrix metalloproteinase (MMP), c-Met receptors and the Rho–Rac interaction, and (d) stimulation of angiogenesis through the release of HIF-1α, vascular endothelial growth factor (VEGF), angiopoietin, and other factors [78]. Cells activate numerous antioxidant pathways that protect them from oxidative conditions. Keap1-Nrf2 is a master antioxidant pathway involved in cytoprotective mechanisms through Nrf2 target gene expression. Activation of the Nfr2 pathway benefits the cells in the early stages and reduces the level of ROS [56,79,80] (Figure 2). The large body of results obtained from extensive studies indicates the special role of the Nrf2 pathway in suppressing tumorigenesis. The activation and translocation of Nrf2 to the nucleus can trigger ARE-related gene expression of a variety of antioxidative and cytoprotective proteins, including heme oxygenase-1 (HO-1), NAD(P)H dehydrogenase, quinone 1 (NQO1), glutathione S-transferase (GST), superoxide dismutase (SOD), glutathione reductase [81], catalase (CA), and some other proteins that can maintain redox hemostasis in cells [82]. In addition to the essential performance of Nrf2 in the upregulation of ARE-related genes, it also boosts antioxidant and detoxification pathways by enhancing the synthesis and reproduction of NADPH. It is used as a cofactor in various redox reactions, such as in GSH reduction by GR, as it can be considered a direct antioxidant [83]. All the antioxidant proteins expressed in the Keap1-Nrf2 pathway remove various types of ROS and RNS. By reducing the level of these active species, they prevent their destructive effects on biomolecules. The structure and function of all these enzymes are fully explained in the previous study of our team [4]. Any defect or disorder in this pathway will lead to tumorigenesis and cancer progression. In addition, Nrf2 and NF-kB pathways, in their unequal parallels, lead to the maintenance of stable cellular redox conditions and inflammation [84]. NF-kB is observed in nearly all types of animal cells and is involved in numerous proceedings such as inflammation, apoptosis, cell growth, and development. A set of subunits that can form a transcription factor dimer of the NF-kB family includes p65, RelB, c-Rel, p50, and p52. These subunits, in the form of heterodimer and homodimer complexes, display multiple functions in the cell. The two most common subunits are p65 (RelA gene) and p50. Commonly, p65/p50 heterodimers act as a transcription activation complex, whereas the p50/p50 homodimer acts as a transcription suppression complex [85]. Under normal conditions, NF-kB subunit dimers are connected to an inhibitory protein known as an inhibitor of NF-kB (IkB) in the cytosol. In contrast, in oxidative stress, activation of IkB kinases (IKK) leads to phosphorylation of IkB and release and nuclear translocation of p65/p50. This process causes the transcription of pro-inflammatory effectors such as interleukin-6 (IL-6), interleukin-1 (IL-1), Tumor necrosis factor α (TNF-α), and inducible nitric oxide synthase (iNOS) [86]. Keap1-Nrf2 plays a key protective role against inflammatory responses (activation of NF-kB) and stimulating the expression of its downstream genes, such as HO-1, can reduce the inflammatory activity of the NF-kB signaling pathway [87,88]. Based on some studies, the activation of the DNA damage response (DDR) has positive feedback on the expression of Nrf2-related genes. The tumor suppressor BRCA1, through the suppression of Keap1-dependent degradation of Nrf2, induces the activation of Nrf2 and promotes Nrf2-target gene expression [89]. Poly [ADP-ribose] polymerase 1 (PARP1), an ADP-ribosyl transferase, is involved in DNA repair by conjugating to BRCA. On the other hand, this enzyme is specified as a coactivator of Nrf2 by linking to Maf-G and increasing the transcriptional activity of Nrf2 [90,91]. Cancer cells display the extreme creation of various proteins rising from epigenetic changes, gene fusion or amplification, and improved metabolic rates. Genomic instability in cancer cells triggers the production of numerous mutated proteins, and their accumulation in cells leads to proteotoxic stresses. Heat shock proteins (HSPs) have the main role of protecting cells against these stresses. HSP is involved in protein folding to degradation systems through the ubiquitin-proteasome system [92,93]. Heat shock factor 1 (HSF1), a transcription factor, is involved in HSPs gene expression. According to some studies, HSPs can trigger Nrf2 to preserve redox balancing and mitochondrial integrity [94]. Furthermore, misfolded proteins in the endoplasmic reticulum (ER) produce similar stress conditions and trigger various pathways. In this condition, unfolded proteins respond via some signaling pathways, including double-stranded RNA (PKR)-activated protein kinase-like eukaryotic initiation factor 2 kinase (PERK) and transcription factor 6 (ATF6) activation. Based on some studies, these signaling pathways, through various mechanisms, can regulate ER stresses and increase cancer cells’ survival against proteotoxic stress [95,96]. A huge number of studies indicate the protective role of Keap1-Nrf2 in cancer. Based on one study, curcumin suppresses the Fen1-dependent proliferation of MCF-7 cells and pointedly prompts Nrf2 protein expression. Therefore, curcumin can prevent the progression of breast cancer cells through Nrf2-mediated down-regulation of Fen1 expression [97]. Based on another study, *N*-(benzylidene)-2-((2-hydroxynaphthalen-1-yl) diazenyl) benzohydrazides (1-2) (NCHDH and NTHDH) increased apoptosis proteins such as Caspase-3, Caspase-9, and Bax, while it repressed B-cell lymphoma 2 (Bcl-2) expression. Moreover, it significantly induced antioxidant signaling proteins such as Nrf2 and Heme oxygenase 1 (HO-1) in the MCF-7 breast cancer cell line [98]. Nymphayol is a sterol terpenoid obtained from the *Nymphaea stellata* wildflower and displays various activities, including anti-inflammatory and anti-proliferative actions. According to this study, increased generation of ROS and its induced apoptosis is the main mechanism of action of Nymphayol in MCF-7 breast cancer cells. Real-time PCR results have shown an increase in mRNA levels of cyclin-dependent kinase inhibitor 2A (Cdkn2a), retinoblastoma protein 2 (pRb2), p53, Nrf2, and caspase-3 [99]. Based on the results, quercetin, kaempferol, and atractylenolide positively affect Nrf2-target gene expression. Upregulation of Nrf2 and its downstream genes, such as NQO1, display a protective role in the MCF-7 breast cancer cell line [100].

## 5. Another Facet of Nrf2, an Oncogene in Cancer Progress

**Figure 3:**
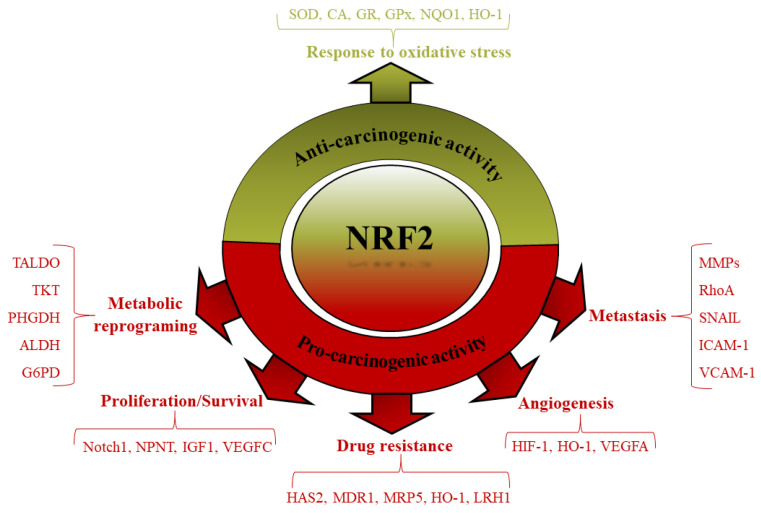
Anti/Pro-carcinogenic activity of Nrf2. Under chronic oxidative conditions, various mechanisms increase cell growth and proliferation in cancer cells. Hyperactivation of Nrf2 is one of the main mechanisms that promote cancer cell proliferation and survival, and increase their resistance against chemo/radiotherapy agents.

As mentioned in the previous section, the anti-proliferative and anti-inflammatory effects of the Keap1-Nrf2 pathway have been proven through strong evidence. In contrast, based on various studies, hyperactivation of the Nrf2 pathway exert an influential role in tumorigenesis through numerous mechanisms [101]. Hyperactivation of Nrf2 has some advantages for cancer cells, including defense against apoptosis and senescence, stimulation of cell growth and development, and resistance to chemo- and radiotherapy [102,103,104] (Figure 3). Some mechanisms are involved in Nrf2 hyperactivation, including (a) somatic mutations in Nrf2 or Keap1, (b) epigenetic modification in the Keap1 promoter, (c) extra accumulation of proteins that disturb the interaction between Nrf2 and Keap1, (d) metabolic reprogramming, and (e) overlapping with other pathways and regulatory miRNAs [42]. All of these mechanisms can trigger hyperactivation of Nrf2 and reduce ROS, creating favorable conditions for the growth and development of cancer cells. As mentioned, Nrf2 targets genes involved in various cellular processes, and its hyperactivation can initiate multiple signaling pathways in the cells. Furthermore, based on some studies, Nrf2 status can affect the rate of cell proliferation, with Nrf2^−/−^ cells proliferating slower and Keap1^−/−^ cells proliferating faster than wild-type cells [105,106,107].

### 5.1. Nrf2 and Cell Proliferation/Survival

Nrf2 regulates various gene expressions involved in cell proliferation and protein synthesis, including Notch1, NPNT, IGF1, VEGFC, PHGDH, PSAT1, PSPH, and SHMT [108,109]. On the other hand, the transcription of Nrf2 increases through some oncogenic proteins, including Kras, Kraf, and Myc, which involve cell proliferation [103,110]. Some specific signaling pathways are involved in cancer cell development, including PI3K/AKT, which correlates with the regulation of Nrf2 target genes [111]. According to the results, Nrf2 regulates the expression of various proteins linked with stemness, such as ALDH enzymes, Notch1, and SIRT1 [112,113,114]. A high level of ROS can promote DNA damage and induce genome instability. On the other hand, the upregulation of DNA repair processes creates cell resistance to various therapeutic agents and promotes carcinogenesis and tumor development. In non-transformed cells, Nrf2 activation can protect cells against oxidative stresses, but in transformed cells, the hyperactivation of Nrf2 is favored for cancer cell survival and proliferation by creating chemo/radioresistance [115,116]. Nrf2 hyperactivation through several mechanisms inhibits genome instability. Nrf2 decreases 7,8-dihydroxy-8-oxo-2′-deoxyguanosine (8-oxo-dG), the main numerous oxidative DNA lesion in DNA, by upregulating 8-oxoguanine DNA glycosylase (OGG1) [117,118].

### 5.2. Nrf2 and Drug Resistance

Downregulation of Nrf2 by inhibiting certain target genes’ expression can induce its differentiation and sensitize them to chemotherapeutic agents [119,120,121,122]. The retinoblastoma protein (RB or RB1), a tumor suppressor protein, is involved in negative cell proliferation regulation. Loss of RB is associated with increased generation of ROS, which is unfavorable for tumor cell progression. Therefore, there is a positive connection between RB deficiency and the inactivation of Nrf2 in cancer cells [123]. On the other hand, Nrf2 activation can provide conditions for cancer cells to escape chemotherapy agent-induced apoptosis. Somatic mutations, epigenetic modifications, and other mechanisms are involved in overexpressing the Nrf2 pathway. Furthermore, Nrf2, through the induction of anti-apoptotic factors such as the Bcl-2 family and decreasing cytochrome c release from the mitochondria and caspase-3/7 activation, suppresses apoptosis in cancer cells under chemotherapy conditions [124,125]. Apoptosis signal-regulating kinase 1 (ASK-1) is activated by oxidative stress and inflammatory cytokines such as TNF-*α*. In normal cells, this kinase is inactive by forming a complex with reduced thioredoxin (Trx); following the increase in ROS and the creation of oxidative stress conditions, this complex is destroyed, and ASK-1 is released and becomes an active kinase [126]. According to new studies, Nrf2 hyperactivation can inhibit ferroptosis, a new kind of cell death relating to iron-dependent lipid peroxidation, by regulating the expression of metallothionein 1G (MT-1G), ferritin and ferroportin to inhibit free iron accumulating [127]. Protection of telomeres is another main mechanism of Nrf2-induced cell survival and decreasing replicative-induced senescence. Senescence is a pivotal event for tumor suppression, and cancer cells can evade death-induced aging. As mentioned, ROS and p53 are related, and different levels of p53 determine their inhibitory/stimulatory effect on the activation of the Nrf2 pathway [128,129]. A cluster of differentiation 44 (CD44), a transmembrane molecule, senses microenvironmental tumor changes by linking to ECM constituents, such as hyaluronic acid (HA), and promotes extracellular signals to control tumor development. A high level of CD44 is related to Nrf2 activation in cancer stem-like cells (CSCs). HA generation was enhanced in doxorubicin-resistant breast cancer MCF7 cells via the upregulation of HA synthase-2 (HAS2). HA can trigger Nrf2 activation and multidrug resistance gene 1 (MDR-1) expression. Therefore, suppressing HAS-2 or CD44 can suppress Nrf2 activity in MCF-7 breast cancer and increase their sensitivity to Doxorubicin [130]. Recent studies have suggested that breast cancer stem cells (BCSCs) play crucial roles in chemoresistance. The role of BCSCs in cancer formation and development, invasiveness, and chemotherapy resistance is becoming rapidly clear. Sex steroid receptors (SSR) can trigger BCSCs proliferation, dedifferentiation, and migration. Estrogen increases Nrf2 activity in MCF7 breast cancer cells through activation of the PI3K/GSK3β pathway. Therefore, estrogen can regulate Nrf2 activity in estrogen receptor-positive breast cancer cells [81,131]. Human epidermal growth factor receptor 2 (HER2) is a member of the epidermal growth factor receptor (EGFR)/HER2 family receptor tyrosine kinases (RTKs). Upregulation of HER2 is related to chemotherapeutic resistance in breast and ovarian cancer cells. According to a study, there is a multilateral overlap in HER2 and Nrf2 activity indirectly. Co-expression of HER2 and Nrf2 promotes Nrf2-target gene expression, such as HO-1 and MRP5. Furthermore, the activity of these two upregulated the mRNA expression of several drug-resistant and detoxifying enzymes. Therefore, as a result, HER2 connects and controls Nrf2-associated transcriptional activation and prompts the drug resistance of MCF-7 cancer cells [132]. Based on a previous study, Nrf2 has a unique interaction with the STAT3 pathway, in which this connection triggers the growth and progression of basal-like breast cancer (BLBC) through transcriptional upregulation of IL-23A expression [133]. L-lysine α-oxidase (LO), a flavoprotein, catalyzes the irreversible oxidative deamination of the essential amino acid L-lysine. This enzyme exerts a unique anti-tumor function due to L-Lys depletion, hydrogen peroxide generation, and the resulting oxidative stress. ROS-induced apoptosis is the main mechanism for LO activity in cancer cells; thus, the knockdown of Nrf2 and its target genes with specific siRNA promotes ROS production and apoptosis in MDA-MB-231 breast cancer cells [134]. Nuclear receptor subfamily 5 group a member 2 (NR5A2, known as LRH-1), an orphan nuclear receptor involved in cell development, migration, and metabolism, has been described to help tumor progression and chemotherapy resistance. The bromodomain and extra-terminal domain inhibitor (BETi) displays remarkable anti-cancer activity in breast cancer. Nuclear receptor coactivator 3 (NCOA3, known as AIB1), as an NCOA3 coactivator, can promote tumor proliferation and development. Based on this study, NR5A2-NCOA3 exert their growth-stimulating role through the inhibition of ferroptosis induced by BETi, by increasing Nrf2 expression and inducing chemotherapy resistance in DA-MB-231 (MB-231), MDA-MB-468 (MB-468), and SK-BR breast cancer cell lines [135].

### 5.3. Nrf2 and Metabolic Reprogramming

Overexpression of Nrf2 upregulated the expression of Notch1 via the G6PD/HIF-1α pathway. Notch1 signaling affected the development of breast cancer cells by affecting its downstream genes HES-1 and p21. The modulation of glucose-6-phosphate dehydrogenase (G6PD)/ HIF-1α expression by Nrf2 is consequently involved in the Notch1 pathway-mediated regulation proliferation, migration, and invasion of breast cancer [136,137]. P21 is a positive regulator of Nrf2, displays a positive feedback regulatory effect on Nrf2 activation in cancer cells, and enhances the viability of cells in oxidative conditions [138]. Furthermore, Nrf2 can increase lifespan and reduce senescence by regulating Notch1 and MDM2, the essential negative regulator of p53 [139]. Therefore, hyperactivation of Nrf2, along with several other mechanisms in cancer cells, causes their immortality [140]. Due to the high metabolism in cancer cells, they need to uptake glucose and metabolize it through the glycolysis pathway. Based on various studies, the activation of Nrf2 plays a pivotal role in metabolic processes in the cancer cell, so hyperactivation of Nrf2 can trigger glucose uptake and deliver it to the pentose phosphate pathway (PPP) to produce ATP and anabolic intermediates required for cell growth and division. For this purpose, Nrf2 induces the expression of various metabolic enzymes, including glucose-6-phosphate dehydrogenase (G6PD) and 6-phosphogluconate dehydrogenase (PGD), transketolase, and transaldolase [141,142]. Furthermore, Nrf2 can control the generation of NADPH, a reducing equivalent involved in the reduction of glutathione and the redox cycling enzymes glutathione reductase (GR) and thioredoxin reductase 1 (TRXR1), confirming the correlation between the anti-oxidant and metabolic activities of Nrf2. Moreover, Nrf2 has a subtle connection to certain signaling pathways, including PI3K/AKT, and triggers the full stimulation of metabolic genes in actively proliferating cells [143,144]. Based on various studies, the metabolism of amino acids and lipids in some steps is controlled by Nrf2. This pathway, by regulating various enzymes involved in related metabolisms, can regulate cancer cell growth and progression [145,146].

### 5.4. Nrf2 and Angiogenesis

Providing oxygen and nutrients to rapidly proliferating cells is one of the limiting aspects of cancer cell development; thus, angiogenesis, the development of new blood vessels, is a pivotal process for tumor cell growth and progression. The hypoxic condition of the tumor cell microenvironment can induce activation of HIF-1α, stimulating the transcription of various growth factors, cytokines, and extracellular matrix (ECM) remodelers to create vasculature [137]. According to previous studies, the downregulation of Nrf2 decreases HIF-1α protein levels and suppresses angiogenesis. On the other hand, in positive feedback, HIF-1α can regulate Nrf2 via ERK1/2 activation through VEGF expression. Moreover, Nrf2 can trigger the expression of some genes, including NQO1, that directly regulate HIF-1α by inhibiting its proteasomal degradation [147,148].

### 5.5. Nrf2 and Metastasis

Metastasis, the spread of malignant cells from a primary tumor to distant sites, is the biggest problem in cancer treatment. Various molecular mechanisms are involved in its creation, such as the epithelial–mesenchymal transition (EMT), invasion, anoikis, angiogenesis, and transport through vessels [149]. Expression of the adhesion protein E-cadherin decrease EMT processes. Nrf2 in cancer cells increases EMT through the downregulation of E-cadherin. Moreover, in cancer cells, increased matrix metalloproteinases (MMPs) such as MMP2 and MMP9 and the release of various growth factors and cytokines cause increased migration and metastasis. Suppression of Nrf2 in these cells can decrease MMP2 and MMP9 gene expression or their activity [150,151]. Anoikis, a mechanism for removing detached cells in physiological or pathological conditions, can trigger ROS generation and activation of the Nrf2 pathway. Cancer cells, through the hyperactivation of Nrf2, can progress in an anchorage-free mode and thus have increased metastatic power [152,153]. Moreover, the induction of Nrf2 increases the expression of glyoxalase 1 (GLO1), which metabolizes the toxic metabolite methylglyoxal (MG) and suppresses anoikis in detached cancer cells [154,155,156]. Furthermore, Nrf2 regulates mitochondrial respiration by providing substrates and controlling the expression of several complex IV cytochrome c oxidase subunits [157,158]. Furthermore, Nrf2 triggers mitochondrial biogenesis via transcriptional activation of PPARɣ and PPARɣ coactivator 1 beta (PGC-1β) [159]. As mentioned, hyperactivation of Nrf2 creates favorable conditions for the growth of cancer cells through the reduction of oxidative stress. NADPH oxidase 4 (Nox4) is a main downstream effector of TGFβ-induced myofibroblast change during fibrosis. Based on a previous study, Nox4 stimulated cancer-associated fibroblast (CAF) survival by stimulating Nrf2 as a leading anti-oxidant modulator. The obtained results have shown the main role of redox signaling via the Nox4-Nrf2 pathway in the tumorigenesis and metastasis of breast cancer cells by stimulating autophagy and survival of CAFs [160]. Hexokinase 2 (HK2), a kinase enzyme involved in glucose metabolism, is prompted in cancer cells and contributes to metastasis. This enzyme increases the level and stability of GSK3 targets, MCL1, Nrf2, and particularly SNAIL, which plays a specific role in the epithelial–mesenchymal transition (EMT) and metastasis [161]. Saxagliptin (Sax) and Sitagliptin (Sit), two common antidiabetic compounds, stimulated murine breast cancer 4T1 metastasis via the Nrf2/HO-1 axis. These agents prompted ROS-dependent nuclear factor kappa B (NF-kB) activation and its downstream metastasis-related gene expression in vitro and in vivo. Activation of the ROS-NF-kB pathway triggers the production of various inflammation cytokines such as IL-6, TNF-α, vascular endothelial growth factor (VEGF), intercellular cell adhesion molecule 1 (ICAM-1), vascular cell adhesion molecule 1 (VCAM-1), IL-1b, and IL-33, as well as some other factors that display a pivotal role in the alteration of the tumor immune-suppressive microenvironment [162]. Based on these findings, targeting Nrf2 and reducing its function in cancer cells may be a functional strategy to reduce the survival of these cells under high oxidative stress and make them sensitive to chemotherapy agents.

## 6. Nrf2 Inhibitors Applications in Breast Cancer Therapy

Based on the above description, hyperactivation of Nrf2 can create a suitable condition for cancer cell growth and proliferation. Furthermore, it induces cancer cell resistance to chemo/radiotherapy agents. Therefore, various inhibitors’ downregulation of Nrf2 in cancer cells can promote ROS-induced apoptosis and cell death. Many studies have focused on the role of Nrf2 inhibitors in preventing several types of cancer. Breast cancer is the second leading cause of cancer deaths among women, accounting for nearly 570,000 deaths in 2015. Over 1.5 million women (25% of all women with cancer) are detected with breast cancer every year in the world [163,164]. The 5-year survival rate of breast cancer patients is above 80% due to early diagnosis and prevention [11]. Several natural and synthetic Nrf2 inhibitors have been studied in breast cancer treatment both in vitro and in vivo.

### 6.1. In Vitro Inhibition of Nrf2 Activity in Breast Cancer Treatment

The functional mechanisms of Nrf2 inhibitors in modulating Nrf2 activity have been identified through several in vitro studies. Based on a previous study, altersolanol B (AB), a minor fungal tetrahydroanthraquinone (THAQ) metabolite, induces apoptosis in MCF-7 cells within the activation of caspase-9 and PARP, the upregulation of Bax, and the downregulation of anti-apoptotic Bcl-2. Furthermore, it down-regulated Nrf2 and its dependent antioxidant genes. Furthermore, it disturbed Akt/mTOR signaling via the downregulation of p-Akt and p-FoxO1 and the upregulation of PTEN [165]. As explained earlier, breast cancer stem cells (BCSCs) play crucial roles in chemoresistance. Luteolin, a flavonoid found in *Taraxacum officinale* extract, decreased the protein levels of Nrf2, heme oxygenase 1 (HO-1), and Cripto-1, which have been specified to support CSC properties [166,167]. Brusatol, a natural agent (quassinoid) obtained from *Brucea javanica*, exerts an anti-cancer property through the downregulation of Nrf2 in various malignant cells. Brusatol activates Nrf2 degradation, reduces cell migration and metastasis, and promotes cancer cell sensitivity to cisplatin. Moreover, it increases G2/M cell cycle arrest and death in MDA-MB-468 and MCF-7 cells with rising concentrations of brusatol (19.5 to 1250 nM). Furthermore, brusatol (2.0 mg/kg) suppressed NADPH quinone oxidoreductase 1 (NQO1) activity 5-fold [168]. Furthermore, brusatol, in combination with trastuzumab, exerts synergistically enhanced anti-tumor activity in BT-474 breast cancer cells. According to this study, trastuzumab increases brusatol-induced ROS accumulation and apoptosis [169]. Ursolic Acid (UA), a pentacyclic triterpenoid carboxylic acid, displays a wide range of biological effects, including anti-oxidative, anti-inflammatory, and anti-cancer properties. Based on the obtained results, UA suppressed the proliferation of MDA-MB-231 cells (EC_50_ = 18.12 ± 1.10 μM). Furthermore, it pointedly suppressed the expression of Nrf2 and its phosphorylated form (phospho S40) p-Nrf2, as well as its target genes, such as NQO1. In contrast, UA does not affect SOD1 expression as a downstream enzyme in the Nrf2 pathway [170]. Artemisinin and derivative antimalaria drugs display anti-tumor activity (IC_50_ value in nM) in several human cancer cell models, including prostate, breast, lung, liver, and acute and chronic myeloid leukemia. These agents can promote ROS generation and inhibit the Nrf2-ARE pathway. Downregulation of the Nrf2 pathway in the MCF-7 Breast cancer cell line induces sensitivity to chemotherapy drugs [171]. Micro-ribonucleic acids (miRNAs), a collection of non-coding RNAs, exhibit a fundamental role in the regulatory mechanism in organisms. The abnormality of the expression of miRNAs is often involved in the incidence of various human diseases, such as breast cancer. Based on the obtained results, the expression level of miR-101 is abnormally decreased in breast cancer tissues; thus, it suggests miR-101 plays a pivotal role in tumor suppression. MiR-101 exerts its anti-tumor activity through the downregulation of the Nrf2 pathway and the induction of ROS production in the MCF-7 cell line [172]. Cardamonin, a chalcone isolated from *Alpiniae katsumadai*, displays anti-inflammatory and anti-tumor activities. According to the results, cardamonin can suppress tumor cell growth and proliferation by inhibiting HIF-1α-mediated cell metabolism. This compound, by decreasing mRNA and protein levels of HIF-1α and downregulating Nrf2, can trigger ROS-induced apoptosis in MDA-MB-231 breast cancer cells [173]. The thiazole-indoline compound ML385, a novel small molecule inhibitor of Nrf2, can downregulate the Nrf2 pathway, leading to increased sensitivity of SUM159 cells to ionizing radiation (IR) [174]. *Pseudomonas aeruginosa* mannose-sensitive hemagglutinin (PA-MSHA) has been described as a novel anti-cancer agent that prompts cell cycle arrest and apoptosis in various human cancer cells. Based on the obtained results, PA-MSHA, through the downregulation of Nrf2 and p62 expression, can suppress MCF-7/MDR growth and proliferation. Down-regulation of Nrf2 and its target genes can sensitize cells to doxorubicin and trigger ROS-induced apoptosis [175]. Moreover, the screening of natural products from marine cyanobacteria displays three known natural products, lyngbyabellin A, grassypeptolide A, and dolastatin 12, which have a remarkable inhibitory effect on Nrf2. Downregulation of Nrf2 and its downstream target genes NQO1, HMOX1, GPX2, and GCLC was identified as a result of 24-h treatment of DA-MB-231 cells with these compounds [176]. *Castanea crenata* (chestnut) leaf extract induces the downregulation of Nrf2 and sensitizes MCF-7 cells to paclitaxel. Co-treatment with the extract and paclitaxel increases the cytoplasmic levels of apoptosis-related proteins and induces apoptosis in MCF-7 cancer cells [177]. Cordycepin, the bioactive compound extracted from ophiocordycipitaceae fungi, shows various anti-tumor properties in cancer cells [178]. This compound, through the downregulation of Nrf2, increases the irradiation sensitivity of MCF-7 and MDA-MB-231 cells. Increasing the level of ROS due to the suppression of Nrf2 is the most important mechanism for increasing the sensitivity of these cancer cells [179].

### 6.2. In Vivo Inhibition of Nrf2 Activity in Breast Cancer Treatment

Because the results obtained from in vitro studies indicate the role of inhibiting Nrf2 activity in inhibiting the growth and proliferation of breast cancer cells, researchers have sought to expand these results through preclinical studies. Various studies have been conducted in this regard, and in this section, we will discuss some of these studies. Erastin is a low-molecular-weight chemotherapy drug that induces ferroptosis in cancer cells. Glycogen synthase kinase-3β (GSK-3β) is an essential molecule in weakening the antioxidant cell response by modulating Nrf2 activity. Decreased expression of GSK-3β was observed in the cancer tissues of breast cancer patients, and Nrf2 was highly expressed in low-GSK-3β-expressed breast cancer tissues. Overexpression of GSK-3β boosted erastin-triggered ferroptosis with elevated ROS and MDA production. This event is reversed by increasing the activity of Nrf2, which results in the growth and proliferation of breast cancer cells. To evaluate the role of GSK-3β expression on erastin-mediated ferroptosis in vivo, GSK-3β overexpression in breast cancer tumor-bearing mice was treated with erastin. The results of this study displayed a significant reduction in xenograft tumor size and a decrease in the expression of Glutathione peroxidase 4 (GPX4) and Nrf2 [180]. Brusatol treatment reduced Nrf2 activity and the activity of Nrf2-target gene NQO1 in tumors in vivo. Cytosolic Nrf2 expression was decreased in brusatol (0.5 mg/kg)-treated animals. The administration of brusatol (2.0 mg/kg) reduced NQO1 activity 5-fold. Furthermore, brusatol treatment in vivo significantly reduced engrafted EAC tumor volume while inducing many more minor changes in body mass than cisplatin [168]. Another study also displayed that brusatol exerts effective growth-inhibitory activity against HER2-positive cancer cells by inhibiting the Nrf2/HO-1 antioxidant pathway and the HER2/AKT/ERK1/2 signaling pathway. Based on the obtained results, the therapeutic efficacy of trastuzumab in combination with brusatol for nude mice bearing established BT-474 and SKOV-3 tumor xenografts was meaningfully more than that of trastuzumab or brusatol injections alone. Trastuzumab, in combination with brusatol, efficiently reverted tumor growth and did not result in marked weight loss [169]. Cordycepin administration in two different xenograft models (pre-irradiation and post-irradiation models), which were injected with MCF-7 (or MDA-MB-231) cells, was performed to validate the effects of cordycepin on the radiosensitivity of breast cancer cells in vivo. In both groups, irradiation exposure reserved the growth and proliferation of breast cancer cells in vivo; however, cordycepin treatment noticeably blocked tumor growth. Based on these results, irradiation exposure via cordycepin administration up-regulated the protein levels of caspase-3, caspase-8, and classic apoptosis markers, signifying that cordycepin sensitizes xenograft breast cancer tumors to irradiation in an animal model. Moreover, examining the amount of Nrf2 protein in the mice from cordycepin combined with the irradiation exposure group displayed the lowest expression of Nrf2 compared with other groups [179]. According to another study, the administration of PA-MSHA as an anticancer agent to MCF-7/ADR-xenografted mice significantly impaired tumor growth by downregulating NRF2 and p62 levels [181]. Caveolin-1 (CAV-1) expression was inversely associated with NRF2 or SOD-2, which could predict the progress of more aggressive forms of cancer. CAV-1 loss prompts SOD-2 upregulation and H_2_O_2_ accumulation, which, in turn, stimulates AMPK activation and, finally, boosts the glycolytic rate of the MCF-7 cells. The increased expression of CAV-1 led to a marked suppression of anchorage-independent growth and repressed the AMPK-dependent activation of the glycolytic switch. For further analysis using a mouse model of induced breast cancer, the reduced expression of CAV-1 triggers the activation of NRF2 and the subsequent upregulation of SOD-2, stimulating an AMPK-dependent glycolytic switch permissible to gaining an extremely highly aggressive state [182]. All in vitro and in vivo studies related to the modulation of Nrf2 in breast cancer are summarized in Table 1. Based on the studies, it can be concluded that targeting Nrf2 may be one of the prominent mechanisms of some drugs, and it could be the basis for the design of novel drugs.

## 7. Conclusions and Future Directions

Nrf2, as a pivotal transcription factor in cells, displays an extensive association with other specific transcription factors and essential signaling pathways. By considering the dual functions of both ROS and the Nrf2 pathway, it is possible to understand a complex network of cellular communication that requires accurate identification, and determining the contribution of each of these pathways in the emergence of new characteristics in cells is of great importance. Upregulation of Nrf2 in cancer cells and its biological compatibility with cancer cells has been determined as a novel mechanism in increasing the unlimited growth and proliferation and creating chemotherapy resistance of these cells. Further studies are underway to confirm these findings. Up/down-regulation of various pathways in cancer cells can directly regulate their ability to proliferate, angiogenesis, metastasis, and multidrug resistance, all essential factors for cancer development. Targeting the Nrf2 pathway and increasing the level of ROS in cancer cells may be one of the goals of chemotherapy agents and increase the sensitivity of these cells to chemotherapy. Extensive studies on the various Nrf2 inhibitors in different cancers and investigations into their different mechanisms of action could be the basis for the design of more effective anti-cancer agents. It is also possible to increase the effectiveness of ancient drugs by reducing the drug resistance of cells.

## Figures and Tables

**Table 1:** Nrf2 inhibitors in breast cancer therapy.

Inhibitor	Model	Mechanism of Action	Ref.
Altersolanol B (AB)	MDA-MB-231 cells MCF-7 cells	- Activation of caspase-9 and poly (ADP-ribose) polymerase (PARP) - Downregulation of PI3K/AKT, NF-κB and ERK1/2 signaling pathways - Downregulation of Nrf2-target genes	[165]
Luteolin	MDA-MB-231 cells	- Downregulation of Nrf2 and HO-1 - Downregulation of sirt3, and Cripto-1 protein expression - Downregulation of stemness related proteins such as Nanog, Oct4, and CD44	[166,167]
Brusatol	MDA-MB-468 cells MCF-7 cells MDA-MB-231 cells MDA-MB-453 cells Tumor-bearing Mice	- Decrease cell viability - Decreases in NQO1 activity - Inhibition of cell migration - Induction of apoptosis - Downregulation of Nrf2 and its target genes	[168,169]
Ursolic acid	MDA-MB-231 cells	- Inhibition of Nrf2 and p-Nrf2 - Inhibition of Nrf2-target gene such as NQO1 - Downregulating Nrf2 via the Keap1/Nrf2 pathway and EGFR/Nrf2 pathway	[170]
Dihydroartemisinin-NHC-Au	MCF-7 cells	- Induction of ROS generation - Downregulation of Nrf2-ARE pathway	[171]
Cardamonin	MDA-MB-231 cells	- Inhibition of cancer cell growth - Suppressing of HIF-1α and mTOR/p70S6K pathway mediated cell metabolism - Induction of ROS production - Downregulation of Nrf2 pathway	[173]
ML385	SUM159 cells	- Inhibition of Nrf2 - Induction of sensitivity to radiotherapy and enhances IR-induced cell death	[174]
PA-MSHA	MCF-7/ADR-xenografted Mice	- Downregulation of Nrf2 and p62 - Inhibition of cell growth and proliferation	[175,181]
Lyngbyabellin A, Grassypeptolide and Dolastatin 12	MDA-MB-231 cells	- Downregulation of Nrf2 pathway and its target genes NQO1, HMOX1, GPX2, and GCLC	[176]
Chestnut	MCF-7 cells	- Downregulation of Nrf2 and its target gene HO-1 - Increase drug-induced apoptotic cell death	[177]
Cordycepin	MDA-MB-231 cells MCF-7 cells Tumor-bearing Mice	- Downregulation of Nrf2 and related genes such as HO-1 - Blocked tumor growth and proliferation - Up-regulation of caspase 3, caspase 8, and classic apoptosis markers - Sensitizing xenograft breast cancer tumors to irradiation	[179]
Erastin	Tumor-bearing Mice	- Reduction of GPX4 and Nrf2 - Reduction in xenograft tumor size - Increased ROS and MDA production	[180]
